# Pattern Recognition: Using Rocks, Wind, Water, Anxiety, and Doom Scrolling in a Slow Apocalypse (to Learn More About Methods for Changing the World)

**DOI:** 10.1177/1077800420960191

**Published:** 2021-09

**Authors:** Annette N. Markham

**Affiliations:** 1 RMIT University, Melbourne, Victoria, Australia

**Keywords:** grounded theory, methodologies, autoethnography, ethnographies, pattern recognition, performance ethnography, sensemaking

## Abstract

In 5 months of COVID isolation, living out of a suitcase in temporary housing, countless fractal patterns emerged. I can’t say if I created these patterns by looking for them, or that I know the whole world by looking at a grain of sand. The truth of the matter is that it feels like the key for massive scale change is just in front of us, but slipping from our grasp. As we move through these days, weeks, and months, we have very little time before the difference recedes again. I address this matter of concern as a matter of method in performative grounded theory piece.

May 10, 2020. Excerpt from my corona diary: Today, I forgot again. I woke, as for the past several days, with a heavy blankness. It doesn’t seem like it’s only inside me, this numb nothing, but extends up and out. Even the sharp punctures of birdcalls in the early morning stillness seem only a repetition of some mechanical alarm clock, tuned out as easily as hitting a snooze button.

Some are calling it “The Great Pause” and this makes sense. But today’s “pause” is not a slowing down or a waiting. It’s a slow drowning by inertia. This is certainly not aided by the repetition of the same day over and over and over, combined with the endless scrolling to find something new. Some news.

This is a different sort of numbness than several weeks ago when we were overwhelmed by the daily, hourly tragedies, forecasts, and fears. That numbness was a much-needed respite from the steady punch of adrenaline from all the more, more, more. Now, as if all the media outlets decided at once that we could only take so many charts and graphs, my news feed is only a steady drip of whatever, whatever, whatever.

Whatever.

Every day it takes a little bit longer to find the energy to think at all. I’m sure it’s just a phase. These ups and downs are typical of coping with continuous stress. One would think it would be dizzying and disorienting. And maybe tomorrow it will be. But for this day, and the last and the last and the last, it is just . . .

***

Right off the bat and not as a footnote, I need to stress that this story comes out of privilege. This is my story of a White, middle-aged privileged Western female, coming undone during a slow apocalypse. It is an old story, one where a person stuck alone in the middle of nowhere, in self- or other-enforced solitude, begins to pick at old wounds, scream into the wind, and face their own humanity.

Under the surface of the vignettes and arguments, you may discern a thread weaving the piece together to advocate strong resistance against normalizing, dehumanizing, and abstracting practices of science, persistent inside both quantitative and qualitative research paradigms. Thus, while this shares my lived experience, it is also a reiteration of a point I have made often about producing ethics through method (Markham, 2005, 2015), as well as a point made often in the journal of *Qualitative Inquiry*: that coming to know something is a political and ethical matter of future making as well as a series of methodological choices. That, as feminists have long held, the personal is political, and, as Denzin (2007) adds, “. . . the political is pedagogical” (p. 467).

This piece emerges from my 5-month intensive saturation in experiencing the pandemic in a state of isolation and liminality; between homes and jobs and continents. It wasn’t on purpose but since it was happening to me anyway, I decided to broaden it into a larger experiment with a group of people drawn to the call to participate in this autoethnographic introspection.^1^

My process throughout was meant to be intense and generative. It combined systematic and rigorous practices of fieldwork alongside moments of playful engagement with nonhuman intimate others and imagined and actual human audiences in what might be best described as performative grounded theory. By creating routines that fostered a state of readiness, I let go, vulnerable to the wind, willing to encounter sadness and despair until I reached saturation. I counted time in daily patterned activities, revisiting the “field site” over and over and over, sampling the material evidence of my wayfinding along the edge of the sea and sky and forest. I was dreamwalking, daydreaming, startled out of the everyday routines of a life I would have characterized as “normal,” and numbed by the sameness of everything that followed. There is^2^ a repetition in solitude that weathers my soul like the wind and water against stone that carves canyons. I sing, whisper, cry, and swallow Sanskrit. I repeat aloud the sentences I say to myself in my head, just to hear my actual voice. This is not an easy piece to compose because I have taken myself to my own wit’s end.

Along the way, I picked up artifacts, sometimes bringing them with me and other times, only making memories of them with a smartphone. I was in a relentless state of constant comparison. With no end in sight. Nothing but this. This, meaning the larger situation of a global pandemic. And also, this, meaning “being with” the moment. In short, this was a situational analysis of the self in solitude, the body in nature, during a pandemic. Not isolated informationally, but in a bubble that included me, innumerable nonhuman companions (in the form of rocks, sand formations, seagulls, waterlines, sea foam, beach trash, masks, three suitcases, rental cars, rabbits, songbirds, changing seasons, countless stars, my mom’s hand knit gloves, red plaid sneakers, my HDMI cable, scarves, and sand dunes), and a global network of people and information, streamed through not just one but four networked devices: a laptop, an iPad, an Apple watch, and a smart phone.

I write this in gratitude and acknowledgment for the contributions of the late Kathy Charmaz, who asked throughout her body of work not just how might we ground our theory, but what purpose should knowledge serve? As she reminds us so often in her work, the remarkable thing about embracing constructivist grounded theory research is that it can take us to unexpected places: “The path may present inevitable ambiguities that hurl you into the existential dislocation of bewilderment. Still, experiences will ensue and your ideas will emerge” (Charmaz, 2006, p. 185).

Indeed. To this, I can only hope that within this piece, I further open spaces where we scrutinize and value the granularity of what grounded theory and critical autoethnography really mean in practice. Even as scholars who read this journal, one that values transgressive and interpretive methodologies, we can sometimes get caught up in the idea that knowing is somehow capturable. Or that we first collect and then analyze data. Or that we are in control of the path from research question to conclusions. Or that our goal is to explain.

But the good researchers I know are in the business of changing the world, at various scales. In these past few months, I have found countless fractal patterns, emerging simultaneously on my screens, in the sky, and on the shore. I can’t say if I created these patterns by looking for them, or that I know the whole world by looking at a grain of sand. The truth of the matter (my truth) is that it feels like the key for massive scale change is just in front of us, but it is slipping from our grasp. And as we move through these days and weeks and months, we have very little time before the patterns recede into the background again. This is a matter of concern,^3^ but also a matter of method. It is precisely in the microscopic that we find the so-called seeds of change.^4^ Or in the case of COVID-19, the urgently needed opportunity to embrace better ways of moving forward as individuals, humans, with other natural, more than human life on this (our only) planet. However, it is also in these micro moments that we forget what we noticed, and the opportunity is lost.

In what follows I first offer some vignettes and snippets of my own lived experience in the past 5 months, characterized under the heading of *The Patterns*. This is followed by some discussion of what this means to me as performative grounded theory in practice, called *The Macro, the Micro, the Political, and the Methodological*. There is an important politic and ethic of telling stories about how we, as humans, try to make different futures by trying to influence seemingly inevitable trajectories. I use the same stories/journal entries to tell the two different tales (borrowing from Wolf’s [1992] *Thrice Told Tale*). They should be read as momentary structures in a game of ‘cat’s cradle’ (to borrow from Donna Haraway), whereby the unpredictable and wild agency of nature and Annette and a global digital media network entangle, but must be tamed into more or less logical or at least readable forms, through the process of re-presentation. They are as much an interpretation of the self as a narrative about the social and personal impact of a global pandemic. And another case for methodological adaptivity and flexibility. And a cry for embracing the immeasurable ethic of trying not to forget, letting go, and making small acts of intervention wherever we can, because we have to have faith that it will make a difference.

## The Patterns

### Patterns of Continuity, Sameness

“Saaa”

“Taaa”

“Naaa”

“Maaa”

These four sounds are central features of my daily life in 2020. Since March 13, I’ve been trying to invite the universe into my being by chanting these ancient Sanskrit words. Many sources for Kundalini yoga and meditation say the sounds comes from one of the oldest mantras *Sat Nam* which means ‘my true essence’ or ‘true identity.’ Although definitions vary, a common interpretation would clarify that

SA is birth, the beginning, infinity, the totality of everything that ever was, is, or will be. TA is life, existence, and creativity which manifests from infinity. NA is death, change, and the transformation of consciousness. MA is rebirth, regeneration, and resurrection which allows us to consciously experience the joy of the infinite. (Eccleston, 2015, n.p.)

Sanskrit as a language was supposedly designed to make changes in our bodies as they enter our ears, or as we shape them in our mouths. As I write these sounds for this article, I can’t help but repeat them aloud or in my head. In the field, these sounds took many different shapes. Sometimes singing, sometimes being swallowed, sometimes gasped through tears, sometimes screamed into the wind. Sometimes floating above my head as I lay in bed listening to the sounds of the night. I’m not sure how this may have de- or re-constructed me.

***

March 21: My head was down, as usual. It seems always down in Denmark,^5^ but especially now. Keep your head down. Keep a distance. Don’t, for goodness sake, meet anyone’s eyes. #detach, #regroup, #socialdistancing #physicaldistancing #stayinplace #stayathome #shelterinplace #stopbeing selfish #bordersclosed.

**Figure 1.**
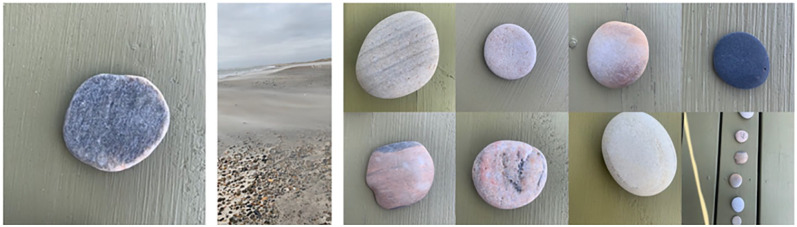
The first rock, a photo of blowing sand in the wind, and seven other rocks I collected during this walk.

I shove my way into the by now gale force winds, and because my progress is so slow, I find myself seeing the most perfectly round rocks. Everywhere. I bend down, pick up a specimen. My left hand rotates and rubs it, satisfying. Yes, this rock is perfect. I fight off an impulse to remove my gloves. I want to get closer to its surface (see Figure 1).

I continue walking, flipping the rock over and over in my palm, in time with my whispering chant:

“Sa, Ta, Na, Ma,”

flip. . . ., flip. . . ., flip. . . ., flip. . . .

My other hand marks the same measure, each fingertip tapping the thumb in a sequence that repeats over and over.

***

Two beats per word, one tap on the first beat.

1 and 2 and 3 and 4 and

SAAA and TAAA and NAAA and MAAA

Tap and Tap and Tap and Tap

Sa . . . Ta . . . Na . . . Ma . . .

**Figure 2.**
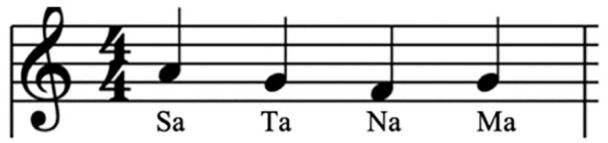
A simple notation of the sound of the Sat Nam chant when sung.

As I walk, I sing this phrase in time to my steps around 375 times (see Figure 2).

I then whisper it to myself another 375 times,

Then I say it silently, shaping the syllables in my mouth, 750 times.

Then I whisper it 375 times

And finally, I sing it 375 times.

Around time 700, if I’m lucky, my head will stop spinning and my mind will start to clear. Around time 1300 (the silent stage, still), I’m desperately bored. When my mouth opens to whisper again, I want to bounce and leap with lightness as the words escape, up and up.

***

Yes, this rock is perfect. Just so.

I almost immediately find another, almost identical, perfectly round flat rock. I pick it up with my right hand. I worry this new rock, turning it this way and that to brush the sand off with my glove.

Keep walking.

Two gloved hands, two rocks, both resembling planets, rotating in my palms.

I stretch my arms wide and lean into the wind.

I am gravity, I think.

Or maybe I’m the sun. Either way, these planets are spinning around me. An idea that matches perfectly my underlying feeling that I’m the center of the universe.

Keep walking. In my ears, covered with noise canceling headphones today to stay warmer, Snatam Kaur chants to me. She was singing, but in the next measure, changes to a whisper. Me too. The sun comes through the clouds now and again, as watery as the tide. The sea is angry. Or at least that’s what I say in my writerly voice. The writer would also say, I think to myself, that the waves are CRASHING against the shore.

Perhaps. But I only hear the smallest small rush of potential noise, as if holding a shell to my ears.

Because I’m in space now. And these rocks are planets.

Each finger is also a planet, I remind myself, in this chant I am dutifully chanting in an attempt at walking meditation.

First finger = Jupiter

Second finger = Saturn

Third finger = Uranus

Fourth finger = Mercury

I try to think about the universe, the big picture, as I walk and chant. I should be tapping fingertips to thumb. But now, I have two rocks. And then four rocks, then six, then seven. All crowded in my hands. They’re all so round today. My gravitational pull must be strong.

Clearly, this meditation is not clearing my head, I think to myself. Still. I continue, into a fierce and bitter winter wind.

This presents a deceptively lovely way of thinking about sensemaking as rhythmic. As the same sounds and rhythms reverberate for hours, days, weeks, there is a sensation of a steadying force, the emergence of a sustained note. Even in the most unpredictable circumstances, a continuity emerges, so quickly routinized as to be eventually unnoticed. It is then simply accomplished, perhaps only recognized when interrupted by moments that mark themselves as moments. Something gets louder, more visible, grows nearer. Something catches our eye, stops our breath, causes us to stumble. These breaks bring their own grammar of exclamation marks, brackets, commas, periods, em dashes of different lengths, ellipses. They punctuate the story. And as I make sense, what are the punctuation marks that interrupt the storyline, the smooth narration? What surprise ends a chapter? What walks through the door? What is important enough to be described?

### Pattern 2: It Looks Like Disrupture, but “Plus ça Change, Plus C’est La Même Chose”

April 8, 2020:

I fight, fight, fight the wind. It feels good. I feel strong. Cold, but strong. But as I turn around and walk away from the face of the wind, I feel shoved. Pushed. It makes me shaky, off center. And now, oddly, I feel weak, nervous, vulnerable. Out of control. Not possible to be detached. The wind seems to mirror my news feeds. Gusting from all sides, forcing me to step in ways that are uncomfortable. Catching me off balance. Ways of being I don’t want, decisions I didn’t ask for. Pushing me too fast toward something and I can’t keep my balance–

Shut in, shuttered,

#closeduntilfurthernotice

we are supposed to weather this storm, ride it out.

#detach #regroup #distance #isolation #recalibration #chinesevirus

The wind hasn’t stopped for days, but it often changes direction. Yesterday, it blew from the East to West. Today, it blows from West to East. I can’t help but use this as a metaphor, around blame. You know, shifting winds, shifting responsibility. (*Chinese Virus? Really? They’re calling it that?!*) The president of the U.S. (my home country) continues to tell citizens they (we) are the exception to the rule.

The winds circle the earth, so whatever went west to east will come back around to bite you in the ass. So my dad used to say. So now, we in the West know better. That no matter how much we push the blame on someone else, the wind will eventually arrive here, in the gusts, borne up my nose. There’s so much irony in the insensitivity of people from different regions accusing each other, playing this game of shifting blame in the midst of a pandemic. A pandemic.

***

**Figure 3.**
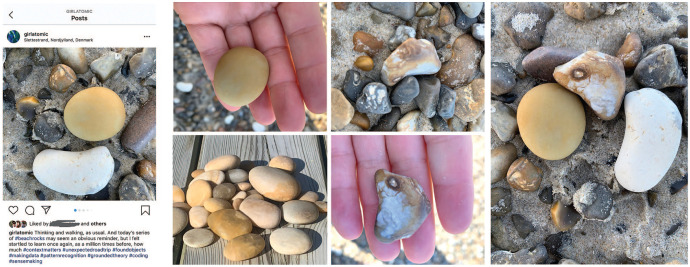
Taking rocks in and out of context to reflect on the complications of sampling.

**Figure 4.**
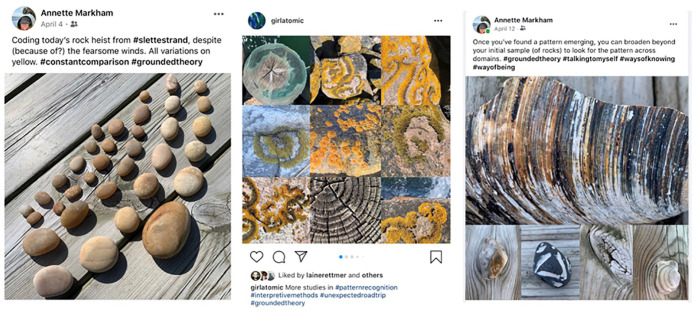
Instagram and Facebook posts using nature as “lessons” in the constant comparison practice of grounded theory.

Meanwhile, in the daily routine of rock collecting, I have noticed that context matters (see Figure 3).^6^ I’m high from all this input and so I have decided to focus on method. My comfort zone. At first, I was rediscovering grounded theory coding, as these posts I share on Instagram and Facebook demonstrate (Figures 3 and 4):

***

June 1, 2020: I’m exhausted. Like someone punched me in the chest. Again today, like yesterday. Probably like tomorrow. Another pattern to recognize. Another element of this situation I’m analyzing. Another moment of comparison. Is this punch like the last one? Or is this a different fist? [They call it doom scrolling, tragedy porn. I call it a global cry for help that if you listen hard enough to, will also punch you. In the chest. Repeatedly]. How can this be simply “grounded theory” or another academic exercise?

***

People are talking about how the experience of the pandemic is coming in waves, as different regions experience the shutting of borders. The United States followed Australia, which followed Denmark, which followed Italy. But these are not gentle waves of social or fashion trends, the sound waves of classical music, or the waves on a calm sea. These are shockwaves, tidal waves, waves of panic washing over us. That’s why it doesn’t feel to me like “wave” at all. It feels more like the wind. An unstable, gusting wind. First, the winds buffet one way, then another. Waves can do this too, but if this is happening to you, you’re likely drowning. I don’t feel like I’m drowning. I am in a swirl of chaos, ups and downs. Moments of calm. Disrupted by yet more news. Again. Again.

***

April 9. Actually, the chaos all feels exactly the same.

**Figure 5.**
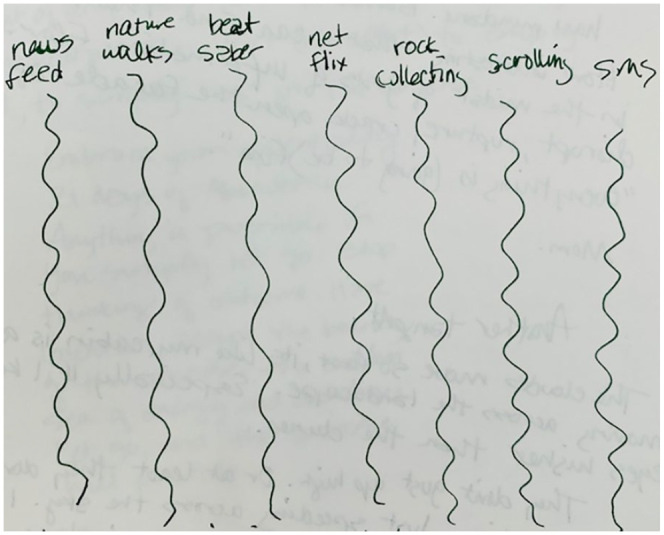
Excerpt from field journal visualizing the sameness across seemingly disparate elements of the everyday lived situation.

There’s literally no distinction between these. News feed is the same as nature walks is the same as Beat Saber is the same as Netflix is the same as rock collecting is the same as scrolling is the same as SMS with family. There’s a dreadful and yet soothing sameness to it, me being moved by the wind, water being pushed again and again onto the shore, scrolling through images on Instagram, time scrolling by as I watch episode after episode of *Battlestar Galactica*. It is the rhythm of crisis in a slow apocalypse. Sometimes passive, sometimes active. Boredom is certainly a part of it, but boredom feels in my body exactly the same as doom . . . just another swell that passes, like my chest as it rises and falls with my breath. Saa. Taa. Naa. Maa (see Figure 5).

**Figure 6.**
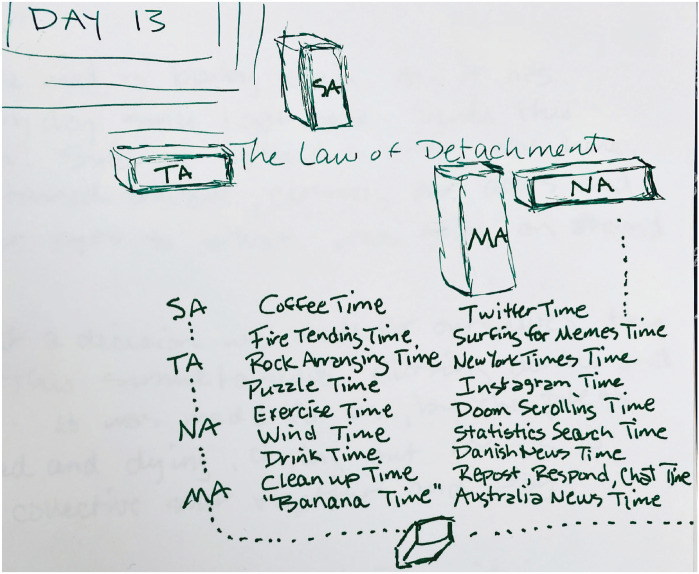
Daily “times..” Repetition. Monotony.^7^

These pulses mark the rhythm of the day, become more evident through endless repetition (only seemingly endless. Just 6 weeks so far). I feel it all with every swipe and click. The mundane, granular, microscopic movements of the day, because they are closed off from normal time, function like a giant mantra. Day long, hour long, over and over (see Figure 6).

**Figure 7.**
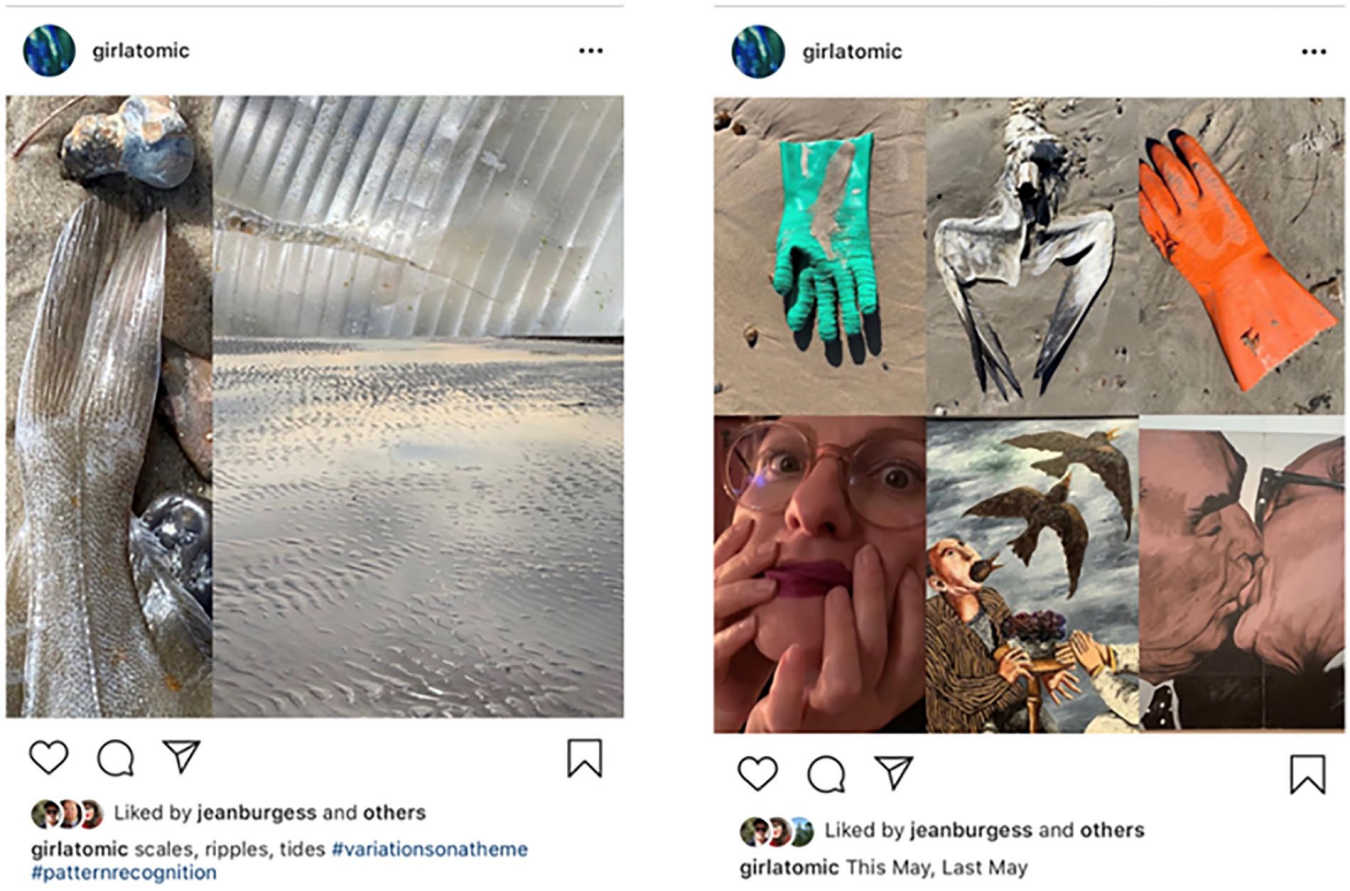
Still finding patterns, still talking about grounded theory in my posts. *Note.* Noticing how I am incorporating trash and dead animals.

They not only punctuate, but, puncture, borrowing the powerful digital poetics of Paul Frosh (2019). Yet when these continuous disjunctures are continuous, they also become a continuity, adding another layer of consistency. This seems to be infecting my image making, as I mix death and garbage into beautiful renderings (see Figure 7).

The question comes to me again and again: What is being punctured? And is it a wound (puncture through the flesh)? Or a release of something pent up (puncture a balloon)? And does it matter if we can’t actually feel it as more than “just more of the same?”

***

“How mundane and banal it all is. I’m completely bored,” I write in my journal.

I immediately write a response, “How extraordinary that you can find this to be merely ‘mundane,’ in the midst of daily—no, hourly!—bursts of information that disrupt, rupture, and practically hit you upside the head with a brick to help you recognize that ‘Everything is (going to be) fine’ is a complete façade.”

I stare out the window. The clouds move so fast it’s like my cabin is a train, rushing across the landscape. Especially if I keep my eyes above the dunes, I can feel my cabin moving.

The wind doesn’t seem to gust when you’re looking up at the clouds. I know differently, of course, having been in many an airplane flying through clouds. But from this distance, it’s just a steady wind moving the clouds across the sky. I think this is what makes me feel like it’s mundane and banal. It’s just time passing. Just time. Passing.

“Just time. Passing,” I say aloud, repeating what I was just saying in my head, “but look at the fireplace.”

I’ve already been looking at the fireplace, since my vocalization of the thought occurs after the thought has already turned my head. This talking to myself business is really starting to annoy me. The gusting wind blows ash out of the fireplace, no matter how tightly I thought I’d closed the flue. It creates a good draw for a fierce fire, but also creates disruptive blasts of wind, pushing flames and cinders down and out. A chaos of disruption, since it occurs quite unexpectedly.

***

Today, the wind stopped. Tonight, actually. I woke up, nervous. I bolted upright, wondering what was wrong. I stared out the window, the gray blue shadows marking time till dawn, not long, not far. A long-legged rabbit hopped into the edge of the scene, paused. Hopped. Paused. Hopped. Then I realized what it was that had startled me awake. The wind was gone.

***

The ache of silence^8^ is still here, many hours later as I write this note. I miss it. The wind. Odd. Its gusting has pushed me off balance so many times in the past weeks. Perhaps it made me access deep cores of my strength to stabilize. Because now that it’s gone, I feel lazy, as if life was too easy. Mind you, this is only a few hours later, so I can’t say this is a meaningful transformation or long-lasting shift. But in the roller coaster of Spring 2020, one must pay attention to these small things. Like this vague thought. Why is it there? Why does the calm feel wrong, a myth? Like it only marks the stagnancy when the flow stops? Or is this just how it is, but we ignore it most of the time?

***

Just moved to a new place. Again. Again, again. Again.

This new place hurts my eyes and ears. Here, the birds sing very loudly. And there is so much green. And the sea is so calm. Together, it all makes me nervous.

I couldn’t figure out why, but then it struck me: Everything here is so *normal*. And I don’t want to go back to normal. I don’t want us to ever go back to whatever we convinced ourselves was normal. I don’t want to be untouched, blythe, disconnected.

I want the wind back.

I want to hear it whistling and I want to howl back at it without regard for how unseemly it must be for Others.

***

**Figure 8.**
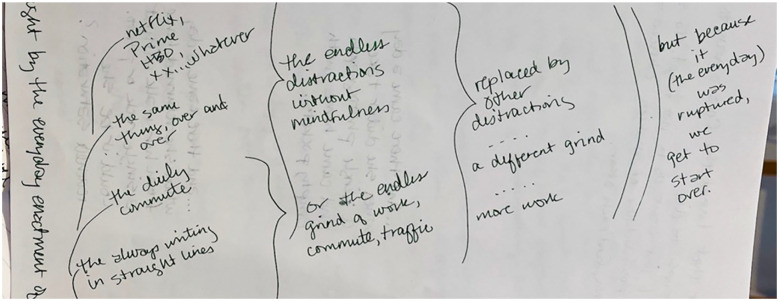
Image from journal visualizing the multiple routines and relations between them that need to be resisted to “be otherwise.” *Note.* Text reads: “The always writing in straight lines, the daily commute, the same thing, over and over. Netflix, Prime, HBO, 1077800420960191, whatever. The endless distractions without mindfulness. Or the endless grind of work, commute, traffic. Replaced by other distractions. . .a different grind. . .more work. But because it (the everyday) was ruptured, we get to start over.

It’s not that I want the coronavirus to continue, or for people to die. I just want us to not forget. To stay aware of the possibilities of making choices to simplify, slow down, find each other. I doodle this, think it through (see Figure 8).

I want us to find oddly appropriate and weirdly serendipitous connections. I want to return to the already fading sense of community brought to me by the urgent need we suddenly felt to be with others, to find meaningfulness in the absence of the mindlessness brought by the everyday enactment of routine.

**Figure 9.**
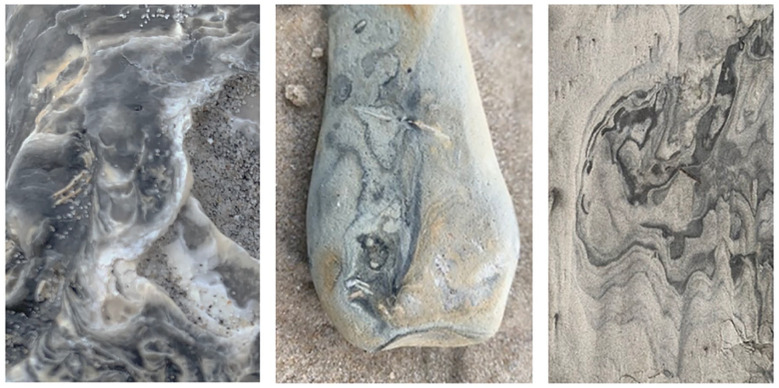
Classic example of finding similarity across different surfaces in my everyday walks. *Note.* Here, from left to right: shell, stone, sand.

How can we preserve an urgency for change, in the midst of the impulse to level or equalize everything? To see regularity and patterns without losing the distinctiveness? Because I feel this urgency for change, but I am drawn to see how everything is so alike (Figure 9).

## The Macro, the Micro, the Political, and the Methodological

This article is, at one level, demonstrating the performative side of grounded theory. Where we work out how the objectivity of the world emerges not from just constant comparison and “themes emerging from the data” we encounter and analyze, but from our noticing and highlighting certain things while suppressing other plausible meanings. And in displaying only some things (in my case in Facebook or Instagram), we perform the calls that seek a response. (#callandresponse; #interaction). I don’t mean to ignore the inherently interactive characteristics of this ongoing social and relational process, but to focus on how a world of choices can get narrowed, seem inevitable. Hence, the article is at another level vivifying and also troubling the potential of recognizing patterns in the microscopic to transform the future, without losing the uniqueness of the granular.

I have spent the past 5 months making my way from one continent to another, in rental cars, with suitcases, from temporary house to another. From late winter in Denmark to spring, the days lengthening visibly as I moved along the coastlines. And then, on the longest day of the year in the far North, I finally flew to Australia to embrace the onset of winter again. Traversing the threads of this cat’s cradle of a year, I shifted from fear to anger, from self-pity to white guilt, from the wide open and endless sand dunes of Danish coastlines to a 2-week quarantine in a Melbourne hotel where I was allowed to leave my room for a 10-min fresh air break once every 4 days.

In many ways, the entire experience has been a deliberate set of actions. To immerse to understand. To obsessively pay attention to and engage actively in the multiple conversations around me, at levels of intensity and reach I didn’t think possible. I wanted to experience what it felt like to *be* in such a time of change and global transformation as an individual, but to also try to put my finger on the pulse of how *cultural change happens* in larger, networked, perhaps global scales. With every heart and thumbs up and retweet and reply, there was and is a larger enactment of what we can hope leads to new patterns.

How can we reshape our frameworks for thinking about the world around us, when the problems seem too massive for our tiny actions and ways of thinking to make a difference? In some ways, it feels like trying to control the impact of the proverbial butterfly flapping its wings. We’re on a planet where even the Arctic is on fire, where violence and brutality are so commonplace we have to watch people die in front of us to even notice something’s wrong. How can we take advantage of the disruptures and anomalies that are breaking the frames^9^ all around us right now?

The performative moves past observation and even beyond sensemaking to generate action in reflexive encounter with others/Other, where small and local sensibilities enter the larger symbolic interaction playing out across global networks. This matters, in that it is a process of what Karen Barad has called “mattering.” That process of not just being with, but becoming with. In this, the most microscopic acts create patterns that can reshape future trajectories on massive scales. In chorus with performance auto/ethnographers like Dwight Conquergood, Tami Spry, and D. Soyini Madison,^10^ I invoke critical reflexivity as a grounding for the performative, to (attempt to) seek or even enable a “we” as a way of encoding different trajectories, ethically better possible futures. Still, as I engage in this, I can’t help but recognize that the very patterns that reveal are the same ones that stall actual, radical, paradigm-shifting transformations.

Physicists, particularly those interested in fractals and small world networks (and here I draw more on Mark Buchanan than Karen Barad), talk of a “power law” for theorizing how patterns emerge out of a stream of historical accidents, creating structures that show remarkable symmetry and self-similarity. Take river (or most types of) networks. As Buchanan (2002) writes,

If you magnify any small portion of a river network, you will get a pattern that looks much like the whole. In other words, the network is not nearly as complex as it appears. Innumerable accidents may make every river network unique, and yet what goes on at one scale is in every case intimately connected to what goes on at another. (p. 103)

Pattern recognition, in recent computational thinking and machine learning, is focused on similarities in data. It’s how Google search for “cat” returns a page full of images of cats. There’s more to it, of course, but the idea behind pattern recognition is that there are arrangements of characteristics that give some information. Most of the time the phrase is used to identify determinate characteristics or tendencies. For a physicist or machine learning specialist, these patterns are not outcomes of looking, but inherent in the data themselves.

Pattern recognition in interpretivist or constructivist grounded theory is something else. There is the same constant comparison of various samples over time, but there is a moment when one chooses which qualities will become obdurate. Pattern recognition makes data. Or to be more specific to the process of science: When we identify the pattern, we make data.

**Figure 10.**
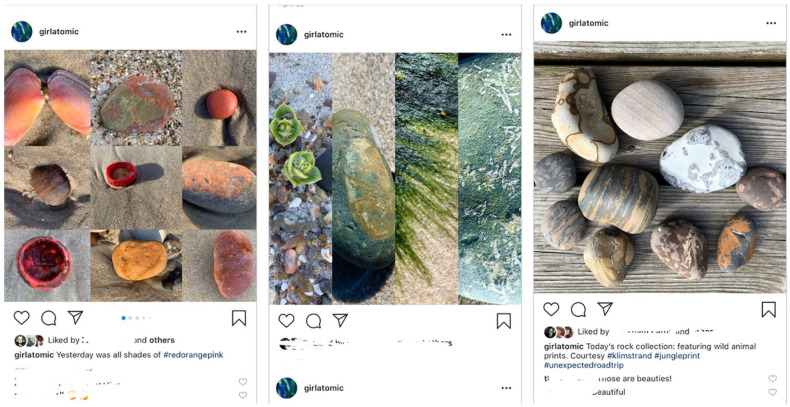
Highlighting the patterns I was seeing for people on Instagram or Facebook.

These two stances operated simultaneously in my world for the past few months. I selected certain patterns to focus on and to share, strategically. One day “red,” the next day “green,” tomorrow “animal prints” (see Figure 10).

**Figure 11.**
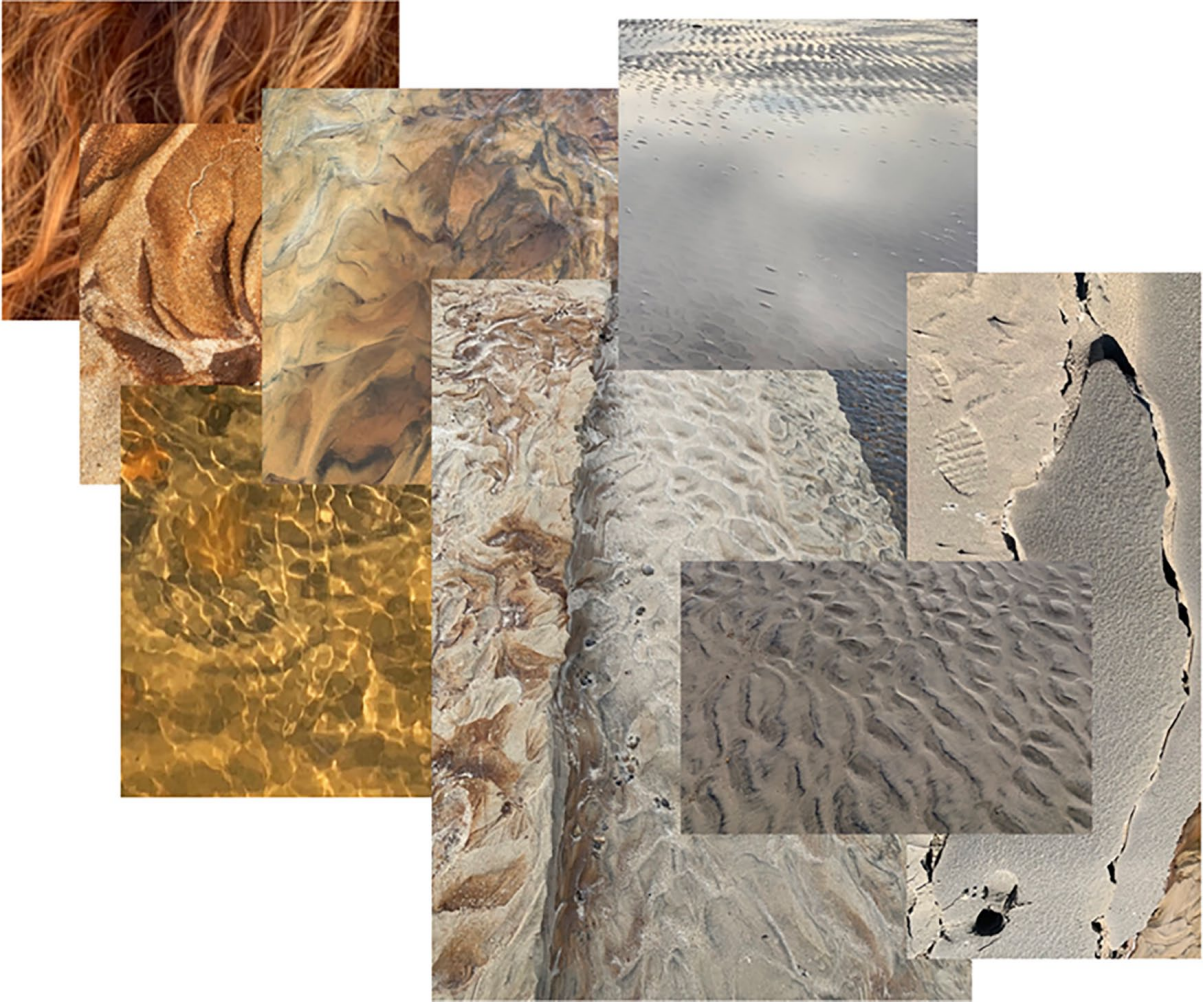
Eight of endless fractals shown here in moodboard format.

Other patterns were just there. Of course, I saw these patterns because I was paying attention (think about Schrodinger’s cat here). But the rhythms of nature were also just there. Undeniably, they kept coming up. And as I combed through thousands of images and many dozens of pages of writing in my COVID diary, I witnessed these fractal patternings over and over, everywhere I looked, even in the mirror (e.g., Figure 11, where one of the images is a close-up of my hair.).

For me throughout this period, despite the fact that my life was disrupted more than most, it was surprising that there was so much repetition. As the surprise fades, it’s clear that these mirroring patterns of sky, water, and sand worked on my sensibilities; began to create the same rhythms in my breathing and stepping. The rhythms then infected my practices of swiping, scrolling. And then they affected my way of writing. And so on.

***

Backing out to the main point again, each microscopic moment, evidenced in each vignette, storyline, shared post, moment of sensemaking, is both a particular and a general. They work together as a part of a larger fractal. Very little is shown in this article, since I have presented only a fraction of what I produced over the past 5 months. Still, even this small sampling can show, remarkably, some common, core sensibilities that arguably (in physics, and the Tao, anyway) give meaning to Whitehead’s phrase “the general in the particular and the eternal in the transitory.” Or Blake’s “to see the world in a grain of sand.” Thus, pattern recognition is both a matter of finding similarities that might already exist, through constant comparison, and also recognizing that there are patterns that are so overwhelming, they must be found to fight against their tendency toward homeostasis at best, entropy and ecological (and social) demise at worst.

## Inevitable Abstractions in Even the Most Grounded of Methods

I draw this piece to a close by reflecting on the inevitable abstraction that occurs because of time, normalizing, and everyday acts of patching together a story to tell someone or the self about “what happened.” Every moment of analytical sense making diminishes the moment of engagement.

Throughout this period, I have tried to remain in a state of defamiliarization, minimizing this inevitability of abstraction. But even in the midst of all this direness, I can’t seem to adequately address what needs to be said. It fades.

***

May 1: “Most of what I wrote in the past six weeks seems banal and self-indulgent.”

***

This is not the first time I’ve written this in my diary. This assessment of everything that has happened over many weeks of COVID isolation is not simply a product of my daily self-criticism or another way of saying that what a White, Western, middle-aged, middle-class person of privilege writes is necessarily and always going to be banal in comparison with what’s happening in the world (#pandemic, #BLM). That’s indeed true, but the theme that seems to be emerging, both the banality and my feeling that it was/is banal, is something to analyze and to be wary of.

From an analytical perspective, these are important signals of shifting sensibilities over time. At this nexus. Whatever I might have felt was meaningful 2 months ago (or 2 weeks ago or 2 days ago) has been superseded by other deeply important matters of concern. As Tressie McMillan Cottom (2020) and her colleagues write, America was “jolted out of its pandemic stupor” (n.p.), by protests that cried loudly and repeatedly for the world to wake up to address “a panorama of structures of oppression.” When I witness the video of the suffocation of a Black man under the knee of a White police officer, my diary statements back in March (see Figure 12) about being “unable to breathe” don’t just take on added meaning, they transform to sign evidence of my privilege in a larger system of racism, of what writer Scott Woods (2014) describes in vivid detail as “an insidious cultural disease” (n.p.). My diary sentiment seemed so important to me when I wrote it. This was back when many of us were obsessed with either *Tiger King* or “flatten the curve” graphics.

**Figure 12.**
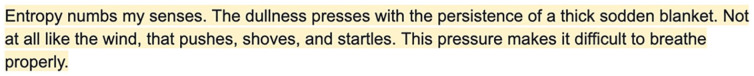
Screenshot from one of many fieldnotes in March and April about breathing, which take on new meaning after #BLM protests.

From a perspective of wariness, my own shifting definition of my diary excerpt above is a reminder that sensemaking is never a one-time thing. Sensemaking is also retrospective,^11^ so any answer to “what happened?” or “what was going on there?” will be an ever-moving target. Why be wary of this? After all, don’t we already know that memories are being continuously overwritten, such that earlier stories are erased? Yet it matters. As countless BIPOC writers such as Rachel Cargle (2020) in the United States or Chelsea Bond (2020) in Australia have reminded us this year, #BLM is precisely an effort to bring violent historical silence and forgetting to the foreground to show and name the damage it has wrought; to “continue to proclaim our presence, everyday and everywhere,” despite “centuries of unbridled colonial violence from a society built upon the idea that we did not exist” (n.p.).

There is a politic as well as an epistemological reason for being reflexive about our choices. And to critically examine why some things count as “valid” in the telling and other things are left out, omitted, not recognized as patterns.^12^

To take this to a different place, in this moment of COVID-19, one of so many local and global traumas, I am wary of the consequences of future collective forgetting and diminishing the importance of the ruptures and critical junctures over time. As the recency fades, many of us will forget. We swing back to what we perceive is normal. This happens despite the continual reminders on our various news feeds that our everyday norms constitute a “new normal.” Even as we cannot return to the prior state of not knowing, we forget that these shifts are radical, were shocking. The same could be said of the months following the 2016 presidential election in the United States, where we promised ourselves and each other that we would not allow the craziness of Trump to become normal. Yet it did.

This normalizing is endemic to ethnographic sensemaking as well. When we answer the question “what is going on here?” we likely recognize that this is not just a where but a when. And this temporality plays tricks on the grounded theorist. Sometimes a fragment, like a coffee stain on a field diary,^13^ can transport the researcher back to the visceral lived moment. The flip side of this knowing is when we can no longer recognize what we wrote because we are no longer in the time/space when it mattered. While I might want to provide some thick autoethnographic description of “how it happens when it happens,” let’s face it: I can no longer find the wind.^14^

***

June 20, 2020. Excerpt from my corona diary.

Where does the banal repetition, the insanity of the whatever whatever sameness come from? Is it only mine? As I write this, i have just forced myself to stop doomscrolling through the newsfeeds.

Slide

Refresh.

Slide finger down

Refresh the screen.

“You are up to date”

. . .

Slide finger down

Refresh the screen.

“You are up to date”

I have been sleeping fitfully, starting awake to the full moon, amazed that it still rises on this broken planet. The steady dose of Dire is overwhelming in these days, and it adds a continuous buzz in the back of my ears. Still, I write. Wanting to write through it. Wanting to find some clarity and meaning. Trying to make sense by letting my fingers flow across the keyboard without much ambition to find a solid thread. Knowing in the back of my head that it will happen anyway. It, meaning the thread and also the ambition and also the sense.

I’m not looking at the screen. Instead, I’m staring out a long, low window into a scene of pure green. The sky shifts from blue to gray in rapid and repeating sequence. The rains and winds sweep across Denmark, pelting fierce, sweeping past. The greens are greener than green could possibly be in real life. I alternately love and hate this beauty. This pastoral landscape, rolling hills of different shades of green, shifting shades along with the direction of the breezes that calmly lift the needles of cypress and ruffle the birch and oak.

I know it is important to chronicle these moments in history. With whatever voice I have (or feel I shouldn’t have but have nonetheless). This, despite the times. And my feelings of white guilt? These feelings are a constant companion to my feelings of guilt about climate change, so I can just add it to the stack of ways I feel sickened by our own actions and inactions. And it is the feeling of many, this spring, in 2020 when the world seems to be falling apart in front of us.

Still, this inner roiling is juxtaposed against birdsong and the intense green of spring shifting to summer. It’s difficult to think with all these birds singing. Truly. All I can think of is rhythm. So much rhythm. Sa. Ta. Na. Ma. ONE and TWO and THREE and FOUR and.

And I can’t help but feel that the pounding of rain and screaming of wind and yelling of birds and crashing of waves and silent steadiness of rocks is a far-beyond-human chorus, repeating a refrain over and over and over. Trying to break through. It’s not something we can hear if we strain too hard. But if we pay attention and breathe, we might learn. To pay attention.

## Notes

1. I am grateful for the entire group of collaborators, but particularly Anne Harris, who decided to come on this journey with me as a co-facilitator of this experiment. Her commitment, intensity, and humor helped form the backbone of this entire special issue.

2. Readers will notice inconsistent tense. This intentionally remains in the text. In other words, my liminality seems to include a blurring between then and now, which I want to represent in the written form. I have edited this article carefully, so these tense shifts are not accidental.

3. This is meant to invoke the concept of shifting from “matters of fact to matters of concern,” a phrase coined by Bruno Latour (2004) that mirrors many recent shifts of lens from observation to intervention. One might read detailed treatises on this by Isabelle Stengers (2015), Karen Barad (2007), and Donna Haraway (cf. 2016), among others. In particular, the sentiment is one that insists that our actions as researchers are sympoietic, not autopoietic, and that if we shift to a new register, this can have marked effect and surprising change across multiple systems (drawing from Haraway’s, 2016 ideas about tentacular thinking, pp. 32–34).

4. Or cuts, as Barad (2007) would say.

5. There’s a longer story behind this sentence. Suffice it to say here that because Danes give each other a lot of personal space, they don’t look at each other when passing by on the streets. There might be a furtive glance, but it’s considered impolite to intrude in the bubble of another person. It may be polite, but it makes me sad and I find I look down and am less socially engaged than when I lived elsewhere.

6. Yes, this is sarcastic.

7. This chart is a nod to the ethnographic work of Donald Roy on the factory floor in the 1950s. In his piece called “Banana Time,” he speaks of remarkable rituals that punctuate the day, providing granular em dashes to break the tedium, a set of parentheses to delineate work from not-work, or perhaps just a bracketing to remind the men that they were live beings with brains and willpower in an otherwise dehumanizing repetition of acts separating mind from hand. Banana time, stealing fruit time, mock fighting time (Roy, 1959).

8. As a sidenote, even as I experienced this, I began intellectualizing this in my journal as “an ache of silence. The stillness exerts an unbearable pressure on my sensibilities.”

9. Here, I am borrowing from Goffman (1974), but one could also use Judith Butler’s work on performativity and gender, Kenneth Burke’s work on language and circumference, Garfinkel’s ideas about social structures.

10. There is a long legacy of work in this area, well represented by Conquergood (1985, 2013), Spry (2011), and Madison (2011).

11. As Karl Weick (1969) memorialized in the phrase “How do I know what I think ‘til I’ve seen what I said.”

12. Drawing on the political premises of critical ethnography and autoethnography, it is necessary but not sufficient to simply be inductive and generative, to find the unexpected, or to recognize that meaning-making is a process of operating in the swirl and flood in the between–what Fine (1994) called ‘the hyphens’ of experience for a moment, to render a temporary knowing for others in what we produce. Rachel Cargle’s (2020) framework of “critical knowledge + radical empathy + intentional action” requires more. As the BIPOC scholars in this special issue (e.g., Lisette Torres, Sun Young Lee, Clair Irwin) and elsewhere remind us, we can do better. Deeper interrogation of the privilege within baseline assumptions about what counts and who counts, even in our most nontraditional and radical forms of knowledge production in the social sciences and humanities, can help us critically examine and change some of the embedded colonizing practices under our taken for granted interpretive methodologies.

13. This image is engrained in my head by Jean Jackson’s (1995) vivid descriptions of ethnographers talking about what happens in and with their fieldnotes.

14. Anyone doing intervention work that seeks to be large-scale or long-lasting can identify how we are thwarted by the frames that persistently build back up, even as we struggle to keep our eyes focused on them. My point here is to point out that even the most profoundly reflexive and activist approaches may not be able to get past this limit, which contradicts what I have often argued (Cf., Markham, 2009) about how reflexivity, local action, and understanding one’s situatedness can help comprise inquiry that has more global sensibilities.
